# miR-214 promotes radioresistance in human ovarian cancer cells by targeting PETN

**DOI:** 10.1042/BSR20170327

**Published:** 2017-07-04

**Authors:** Qin Zhang, Shuxiang Zhang

**Affiliations:** Department of Oncology, The First People’s Hospital of Jining, Shandong 272011, China

**Keywords:** miR-214, ovarian cancer, PTEN, radioresistance

## Abstract

Ovarian cancer is one of the leading causes of death among gynecological malignancies. Increasing evidence indicate that dysregulation of microRNAs (miRNAs) plays an important role in tumor radioresistance. The aim of the present study is to investigate whether microRNA-214 (miR-214) was involved in radioresistance of human ovarian cancer. Here, we showed that miR-214 was significantly up-regulated in ovarian cancer tissues and radioresistance ovarian cancer cell lines. Transfection of miR-214 agomir in radiosensitive ovarian cancer cell lines promoted them for resistance to ionizing radiation, whereas transfection of miR-214 antagomir in radioresistance ovarian cancer cell lines sensitized them to ionizing radiation again. Furthermore, we found miR-214 effectively promoted tumor radioresistance in xenograft animal experiment. Western blotting and quantitative real-time PCR demonstrated that miR-214 negatively regulated PTEN in radioresistance ovarian cancer cell lines and ovarian cancer tissues. Taken together, our data conclude that miR-214 contributes to radioresistance of ovarian cancer by directly targeting PTEN.

## Introduction

Ovarian cancer is the fourth most frequent fatal cancer in women and the leading cause of death from gynecological malignancies [1,2]. The majority of patients with ovarian cancer are diagnosed with advanced disease. The modern management of ovarian carcinoma is the aggressive surgical removal of tumor masses, chemotherapy, and radiotherapy [3]. Despite significant advances in primary treatment, the majority of patients with an advanced stage ovarian cancer ultimately suffer disease recurrence [4–7]. A common problem limiting treatment success as well in chemotherapy as in radiotherapy is the development of resistance [8]. Despite the knowledge that has been accumulated over decades, the mechanisms of ovarian cancer resistance are not fully understood. Therefore, it is of great clinical value to study the molecular mechanism of ovarian cancer resistance.

MicroRNAs (miRNAs) are a class of endogenous short noncoding RNAs that suppress gene expression through base pairing with the 3΄-untranslated region (3΄-UTR) of target mRNAs, leading to post-transcriptional inhibition or mRNA degradation [9]. It was recently determined that microRNAs may play a role in the cellular response to radiotherapy [10,11]. miR-214 is an important miRNA that has been investigated in various tumor types and aberrantly expressed in various cancers and involved in the progress of malignant tumors [12–16]. miR-214 has been previously shown to promote the chemoresistance of human ovarian cancer [17,18]. However, the role of miR-214 in the radiotherapy of ovarian cancer and the molecular mechanisms by which miR-214 exerts its functions remain largely unknown.

In the present study, we found that miR-214 levels are increased in primary ovarian cancer tissues and radioresistance ovarian cancer cell lines. miR-214 enhanced the resistance of ovarian cancer cell lines to radiation, while inhibition of miR-214 sensitized radioresistance ovarian cancer cell lines to radiation treatment. There results were further confirmed by xenograft animal experiments. We identified PTEN as a direct functional target of miR-214 for the induction of radioresistance. These findings suggest that ionizing radiation induces expression of miR-214 in ovarian cancer, which targets tumor suppressor PTEN and consequently activates the PI3K/Akt pathway, leading to increase in ovarian cancer radioresistance.

## Materials and methods

### Patients’ specimens

Matched ovarian cancer specimens and adjacent normal tissues were obtained from 30 patients who had been treated with radiotherapy in The First People’s Hospital of Jining (Shangdong, China). Written informed consent was obtained from every study participant. The present study was approved by the Ethics Committee of The First People’s Hospital of Jining.

### Cell culture and ionizing radiation

Human ovarian cancer cell lines SKOV-3 and IOSE-80 were purchased from the American Type Culture Collection (Manassas, VA, U.S.A.). Cells were cultured in Roswell Park Memorial Institute (RPMI)-1640 medium supplemented with 10% (v/v) fetal bovine serum (FBS; HyClone, U.S.A.) in a humid wet atmosphere containing 5% CO_2_ at 37°C.

To generate a radioresistant cell line, we exposed human ovarian cancer cell line five times in exponential growth phase to a dose of 6 Gy radiation. An interval of 4 to 6 weeks between each radiation allowed the surviving cells to regenerate. The radioresistant cell lines were named SKOV-3/RR and IOSE-80/RR respectively.

### RNA extraction and quantitative real-time polymerase chain reaction

Total RNAs or miRNAs from cancer specimens or cells were extracted using an RNeasy kit or miRNeasy mini kit (QIAGEN, Dusseldorf, Germany) respectively, according to the manufacturer’s instructions. qRT-PCR was performed to detect miRNAs expression using TaqMan® miRNA reverse transcription kit and TaqMan miRNA assay kits (Applied Biosystems), following the manufacturer’s protocol. Expression levels for each gene were normalized to that of rRNA U6 and then converted into relative values calculated by the comparative *C*_T_ method. qRT-PCR was also used to measure mRNA expression with TaqMan® miRNA reverse transcription kit and sybergreen supermix (Biorad). GAPDH mRNA was used as an internal control to normalize PTEN mRNA level.

### Cell line construction and transfection

Virus particles were harvested 48 h after pLVX1-shmiR-214-puro cotransfection with the packaging pRSV/pREV, pCMV/pVSVG, and pMDLG/pRRE into 293T cells using Lipofectamine 2000 reagent (Invitrogen). SKOV-3 cells were infected with recombinant lentivirus plus 8 mg/ml polybrene (Sigma, St. Louis, Missouri, U.S.A.). Cells were grown in the presence of 6 μg/ml puromycin for selection of stably transfected clones. Single clones were picked to generate monoclonal cell lines and named SKOV-3-shmiR-214. The empty lentiviral vector was used as a control.

The agomir-214 and antagomir-214 were synthesized by Sangon Biotech, Shanghai, China. The transfection was performed using Lipofectamine 2000 (Invitrogen, Calsbad, CA, U.S.A.), according to the manufacturer’s instructions. SKOV-3, SKOV-3/RR, IOSE-80, and IOSE-80/RR cells were plated at a density of 5 × 10^3^ cells per well in a 96-well plate. Twenty-four hours after plating, cells were transfected with 40 nM agomir-214 or antagomir-214.

### Survival foci formation assay

Cells in exponential growth phase were plated into a 6-well plate at 3,000 cells/well and then incubated with 40 nM agomir-214 or antagomir-214. Twenty-four hours after transfection, cells were treated with the indicated IR dose. When most cell clones had reached >50 cells, they were stained with 0.06% Crystal Violet.

### Apoptosis assay

Cells in exponential growth phase were plated into a 6-well plate at 1 × 10^6^ cells/well and then incubated with 40 nM agomir-214 or antagomir-214. Twenty-four hours after transfection, cells were treated with the 6 Gy IR. Twenty-four hours post-IR, the cells were trypsinized, washed in PBS, and fixed in ice-cold 70% ethanol overnight. Fixed cells were collected by centrifugation, washed in PBS, and resuspended in Annexin V-fluorescein isothiocyanate (FITC), propidium iodide (PI) (Becton Dickinson, NJ, U.S.A.) for 25 min at 37°C in the dark. The cells were analyzed by flow cytometry (BD CantoII). FACS data were analyzed using FlowJo (Tree Star, Inc.).

### Tumor xenograft experiments

SKOV-3-shNC and SKOV-3-shmiR-214 cells (5 × 10^6^) were injected subcutaneously into 5-week-old BALB/C nude mice. The tumor volume was measured with a caliper once every 4 days using the following formula: *V* (mm^3^) = 0.5 × length × width^2^. When the tumor volume reached 200 mm^3^, it was irradiated with a single 10 Gy dose. Tumor size was calculated every 4 days.

### Luciferase reporter assays

The DNA oligonucleotide and the pMiR-Reporter Vector were used to build the luciferase report vectors (pMiR-PTEN-WT, pMiR-PTEN-Mut). SKOV-3 and SKOV-3/RR cells were transfected with pMiR-Reporte, pMiR-PTEN-WT, and pMiR-PTEN-Mut. A Renilla luciferase-expressing plasmid pRL-TK (Promega) used as control was also cotransfected. Cells were harvested and luciferase activity was determined using the Dual Luciferase Reporter Assay Kit (Promega) at 24 h after transfection. The results are expressed as relative luciferase activity (firefly luciferase/Renilla luciferase).

### Western blot analysis

Total cell protein extracts were separated by SDS/10% polyacrylamide gel electrophoresis, and transferred onto a polyvinylidene difluoride membrane (Millipore, U.S.A.). The membrane was blocked for 1 h in PBST with 5% non-fat milk at 4°C. Then, the blots were incubated with primary antibodies against Akt (SantaCruz), p-Akt (Ser473) (SantaCruz), PETN (SantaCruz), GAPDH (Cell Signaling) followed by horseradish peroxidase-conjugated secondary antibody and detected by chemiluminescence detection kit (Millipore, Billerico, Massochusatts, U.S.A.).

### Statistical analysis

Statistical analysis was performed using a SPSS software package (SPSS Standard version 13.0, SPSS Inc.). Data were expressed as mean ± standard deviation (SD) of at least three independent experiments. Data were considered to be statistically significant when *P*<0.05(*), *P*<0.01(**), and *P*<0.001(***).

## Results

### miR-214 expression is up-regulated in ovarian cancer patients and radioresistant ovarian cancer cell lines

**Figure 1:**
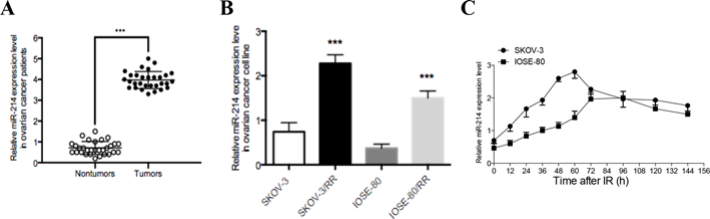
miR-214 expression is increased in ovarian cancer patients and radioresistant ovarian cancer cell lines (**A**) qRT-PCR analysis of miR-214 expression in 30 cases of ovarian cancer specimen and paired non-tumor tissues (*n*=30). (**B**) qRT-PCR analysis of miR-214 expression in ovarian cancer cell lines including SKOV-3, SKOV-3/RR, IOSE-80, and IOSE-80/RR. (**C**) miR-214 expression was detected by qRT-PCR in SKOV-3 and IOSE-80 ovarian cancer cell lines at the indicated time following a 6 Gy dose of IR. U6 served as an internal control. Each experiment was performed in triplicate; ***P<0.001. Data were presented as mean ± SD.

**Figure 2:**
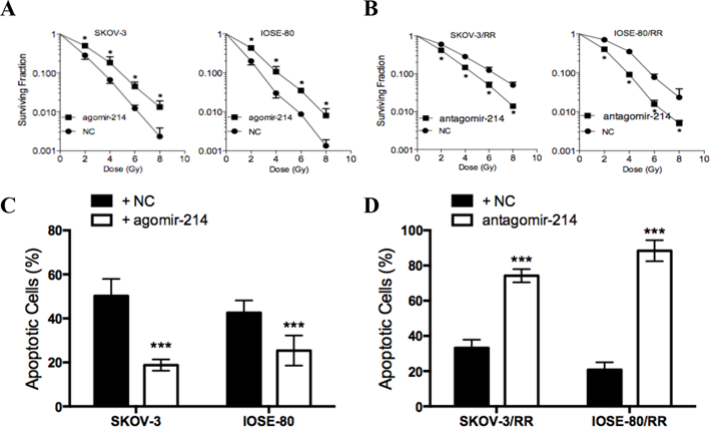
miR-214 promotes radioresistance of ovarian cancer cell lines (**A**) Surviving fraction of parental ovarian cancer cells after the indicated doses of IR. (**B**) Surviving fraction of ovarian cancer RR cells after the indicated doses of IR. (**C**) The percentage of apoptotic cells in parental ovarian cancer cells treated with agomir-214 or agomir-NC. (**D**) The percentage of apoptotic cells in ovarian cancer RR cells treated with antagomir-214 or antagomir-NC. Each experiment was performed in triplicate; ***P<0.001, *P<0.05. Data were presented as mean ± SD.

To investigate the biological role of miR-214 in ovarian cancer, quantitative real-time PCR was initially performed to measure miR-214 expression levels in 30 ovarian cancer tissue samples. As shown in Figure 1(A), the expression of miR-214 was also substantially elevated in ovarian cancer tissues when compared with that in the corresponding non-tumor tissues. To simulate the clinical scenario of radioresistance, we established two radioresistant (RR) ovarian cancer cell sub-clones derived from SKOV-3 and IOSE-80 through repeated exposure of the parental cells to IR. We compared miR-214 expression in ovarian cancer radioresistant subclones and their parental cell lines, and the results showed that miR-214 expression was increased by 3.1-fold and 4-fold in SKOV-3/RR and IOSE-80/RR cells respectively (Figure 2B). miR-214 was significantly increased in these cells as early as 12 h after IR, reached maximum expression at 60–72 h, and then decreased to a stable expression level (Figure 2C). These findings indicate that ovarian cancer cells up-regulate miR-214 expression in response to IR.

### miR-214 promotes radioresistance of ovarian cancer cells

In order to examine the effect of miR-214 on radioresistance in ovarian cancer cells, we elevated miR-214 expression level by transfecting miR-214 agomir into parental cells and decreased miR-214 levels by transfecting miR-214 antagomir into RR cells. Cell survival upon IR showed that miR-214 overexpression induced radioresistance in parental cells (Figure 2A), while miR-214 inhibition significantly decreased the surviving fraction of RR cells post-IR (Figure 2B). As it indicated in Figure 2(C) and (D), under the exposure of 6 Gy IR, miR-214 overexpression significantly reduced apoptotic cells in parental cells, while miR-214 inhibition increased apoptotic cells in RR cells. Combined with the results of radiobiological parameters, these findings indicate that miR-214 promotes radioresistance *in vitro* and that decrease expression of miR-214 might possess radiosensitive potential.

### miR-214 promotes radioresistance in nude mice

**Figure 3:**
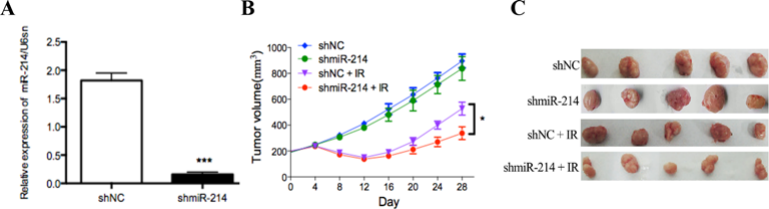
miR-214 promotes radioresistance of SKOV-3 cells *in vivo* (**A**) miR-214 expression was detected by qRT-PCR in the shmiR-214 and shNC groups. (**B**) and (**C**) Nude mice were subcutaneously injected into the posterior flank with 5 × 10^6^ cells infected with shmiR-214 or shNC (*n*=5); ***P<0.001, *P<0.05. Data were presented as mean ± SD.

To confirm the radiosensitive effect of miR-214 inhibition *in vivo*, we infected SKOV-3 cells with lentiviruses encoding shmiR-214 or control shRNA. miR-214 expression was detected by qRT-PCR (Figure 3A). Subsequently, SKOV-3-shmiR-214 and SKOV-3-shNC cells were inoculated into the flanks of nude mice to establish subcutaneous xenografts that were then treated with a 0 or 6 Gy dose of IR. In the absence of IR, tumor growth was slightly decreased upon miR-214 inhibition. However, after exposure to IR, the SKOV-3-shmiR-214 tumor growth was dramatically slower than that of the control tumors. At 28 days after radiotherapy, SKOV-3-shmiR-214 tumor volumes were diminished by 62.3% versus 41.1% in SKOV-3-shNC tumors (Figure 3B). These data suggest that miR-214 inhibition sensitizes ovarian cancer cells to irradiation treatment. In other words, miR-214 promotes radioresistance in ovarian cancer cells to irradiation treatment *in vivo*.

### PTEN is a direct target of miR-214

**Figure 4:**
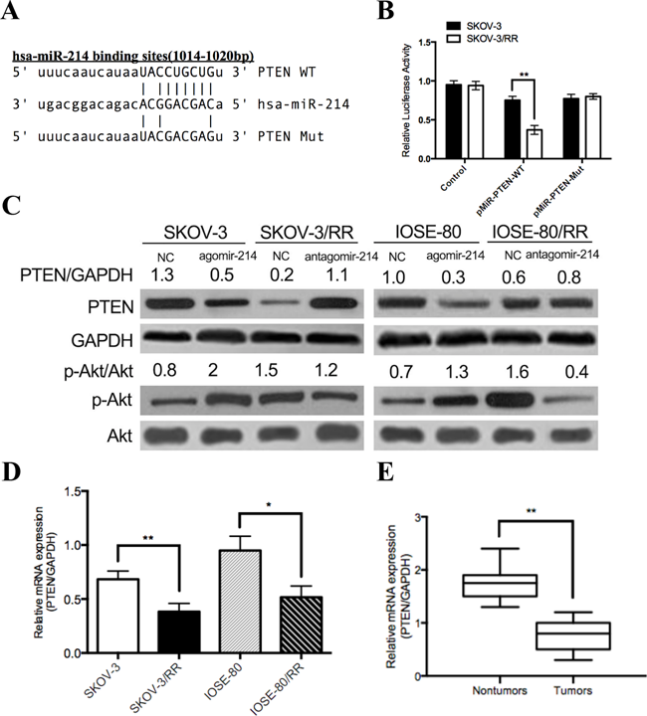
PTEN is a direct target of miR-214 (**A**) Wild-type or mutant miR-214 target sequences in the human PTEN 3′-untranslated region (3′-UTR). (**B**) Luciferase reporter assay of wild-type or mutant PTEN 3′-UTR reporter. (**C**) Western blotting analysis of PTEN and Akt expression in ovarian cancer cells. (**D**) Relative PTEN mRNA expression in ovarian cancer cells. (**E**) Relative PTEN mRNA expression in tumor specimen. Each experiment was performed in triplicate; **P<0.01 and *P<0.05. Data were presented as mean ± SD.

To explore the molecular mechanism of miR-214, we adopted bioinformatic algorithm, miRanda, to predict many potential miR-214 target genes. Among them, PTEN was found to have a putative miR-214-binding site within its 3’-UTR (1014–1020 bp) (Figure 4A). To verify whether PTEN is a direct target of miR-214, WT or Mut 3΄-UTR of PTEN was inserted into the downstream of the firefly luciferase gene. Then, the SKOV-3 and SKOV-3/RR cells were transfected with pMiR-Reporter, pMiR-PTEN-WT, and pMiR-PTEN-Mut. As shown in Figure 4(B), luciferase activity was significantly decreased in pMiR-PTEN-WT group in SKOV-3/RR cells, while it had no significant effect on pMiR-PTEN-Mut group. In addition, Western blot analyses showed that compared with the parental cells, RR cells showed a clear attenuation of PTEN protein expression, which was negatively correlated with miR-214 expression. Furthermore, overexpression of miR-214 decreased PTEN expression in parental cells, whereas inhibition of miR-214 expression significantly restored PTEN protein expression in RR cells (Figure 4C). To further confirm the relationship between miR-214 expression and PTEN, we found that PTEN mRNA was decreased in RR cell lines and in tumor specimen, which was negatively correlated with miR-214 expression level (Figure 4D and E). These results indicate that PTEN is a direct target of miR-214 in ovarian cancer cells.

## Discussion

Aberrant changes in miRNA profiling are implicated in almost all aspects of cancer biology, including tumor development and malignant transformation [19]. Thus, miRNAs are increasingly regarded as a potential diagnostic and therapeutic tool [20,21]. Growing evidence has suggested that dysregulation of miRNAs contributes to tumor radiosensitivity [22–25]. In the present study, we focused on miR-214, which displays complex and contrary behavior in ovary cancers. miR-214 is overexpressed in ovary cancers and significantly associated with high grade and late/metastatic tumor stages, as well as with overall and progression free survival and post-surgical/chemotherapy recurrence [17,26,27]. However, miR-214 down-regulation has also been reported in neoplastic, as compared with normal ovaries, as well as in ovary cancer-derived effusions as compared with primary tumors [27–29]. Therefore, the role that miR-214 may play in ovarian cancer radiotherapy needs to be further elucidated.

miR-214 is deregulated in a number of human cancers including ovarian, breast, melanoma, gastric, and hepatocellular carcinomas. miR-214 participates in the control of cancer cell signaling networks and exhibits controversy functions via regulating several target genes. In fact, miR-214 behaves as a key hub not only by coordinating fundamental signaling networks such as PTEN/AKT, β-catenin, and tyrosine kinase receptor pathways, but also by regulating the levels of crucial gene expression modulators: the epigenetic repressor Ezh2, tumor suppressor p53, transcription factors TFAP2, and another microRNA, miR-148b [17,27,30–35]. Thus, miR-214 seems to have essential roles in coordinating tumor proliferation, stemness, angiogenesis, invasiveness, extravasation, metastasis, and drug resistance.

In the present study, we provide the first evidence that miR-214 promotes ovarian cancer radioresistant by targeting PTEN. Our results indicate that miR-214 significantly increased in ovarian cancer patients’ tumor specimen who had been treated with radiotherapy. miR-214 was induced shortly after the exposure of radiation in ovarian cancer cell lines and this change seemed to be established intrinsically. Specifically, we found that the overexpression of miR-214 significantly rendered the radiosensitivity cell line immune to radiation, while inhibition of miR-214 significantly increased radiosensitivity to ovarian cancer radioresistant cell lines. What’s more, blocking endogenous miR-214 in SKOV-3/RR cells decreased the tumor volume *in vivo* after IR. Eventually, we identified that miR-214 directly targeted PTEN and miR-214 expression level was negatively correlated to PETN expression.

PTEN (also known as MMAC-1 or TEP-1) is one of the most frequently mutated tumor suppressors in human cancer and is an important regulator of proliferation, differentiation, and apoptosis [36]. PTEN is a negative regulator of PI3K/AKT pathway, thereby being involved in the regulation of apoptosis, DNA damage repair and EMT during embryonic development, cancer progression, and radiotherapy [37–39]. Previous study reported that microRNA-214 acts as a potential oncogene in breast cancer by targeting the PTEN-PI3K/Akt signaling pathway [40]. To further explore the molecular mechanisms of tumor radioresistant induced by miR-214, we examined the expression of Akt. The results suggested that the p-Akt was positively correlated with miR-214 expression, indicating that miR-214 may be an important up-stream regulator of this signaling pathway. All these results documented that miR-214 directly targeted PTEN, leading the degradation of PTEN mRNA, which in turn, activated PI3K/Akt pathway and promoted tumor radioresistant of ovarian cancer cells.

In conclusion, the present study provides novel evidence that miR-214 promotes ovarian cancer cell radioresistant through repression of PTEN and activation of PI3K/Akt pathway. Our findings on miR-214 are encouraging and suggest that this miRNA could be a potential target for the treatment of ovarian cancer in future.

## Abbreviations

EMT: Epithelial Mesenchymal Transition
IR: Ion Radiation
PTEN: Phosphatase and tensin homolog
PI3K: Phosphoinositide 3-Kinase
